# NAFLD accelerates arterial perfusion in rabbit VX2 liver tumors: a sonoVue-based contrast-enhanced ultrasound study

**DOI:** 10.3389/fonc.2025.1638869

**Published:** 2025-09-09

**Authors:** Zhi-long Liu, Qin Lu, Qian Zhang, Wen-wen Fan, Juan Chen, Li-ping Liu

**Affiliations:** ^1^ Department of Ultrasound Intervention, First Hospital of Shanxi Medical University, Taiyuan, Shanxi, China; ^2^ The Fifth Clinical Medical College of Shanxi Medical University, Taiyuan, Shanxi, China

**Keywords:** non-alcoholic fatty liver disease(NAFLD), VX2 tumor, contrast-enhanced ultrasound(CEUS), microcirculation, microvessel density(MVD)

## Abstract

**Objective:**

To investigate whether the background of non-alcoholic fatty liver disease(NAFLD) affects the microcirculation characteristics of VX2 hepatomas, analyze the influence of the fatty liver background on tumor angiogenesis and proliferative activity.

**Methods:**

24 rabbits were used to establish VX2 tumor models in the background of normal liver and NAFLD. Contrast-enhanced ultrasound was performed using SonoVue. The time-intensity curve(TIC) parameters were extracted and the expression levels of microvessel density(MVD), vascular endothelial growth factor(VEGF), Ki-67 and PCNA in tumor tissues were detected and analyzed.

**Result:**

The TIC parameter arrival time(AT) and rise time(RT) of VX2 liver tumors decreased under the background of NAFLD(*P*<0.05). There was no statistically significant difference in the tumor size, MVD, VEGF, Ki-67 and PCNA expression levels between the NAFLD group and the normal liver group(*P*>0.05). PI was positively correlated with MVD in both normal liver(r=0.51, *P*<0.05) and NAFLD(r=0.67, *P*<0.05) backgrounds.

**Conclusion:**

The background of NAFLD leads to an increase in the arterial flow velocity of VX2 tumors in the liver, but it has not significantly affected the level of angiogenesis and proliferation. The CEUS parameters can effectively reflect the level of tumor microangiogenesis and are effective tools for evaluating microvessels.

## 1 Introduction

The invasive growth and metastasis process of tumors are highly dependent on tumor angiogenesis and cell proliferation (1, 2). Microvessel density(MVD) is the gold standard for assessing tissue angiogenesis (3), Vascular Endothelial Growth Factor(VEGF) is a key regulator of tumor microangiogenesis (4), Ki67 and PCNA can reflect the proliferative activity of tissues (2), which are closely related to tumor aggressiveness, metastasis and prognosis (5). However, the above tests rely on pathological tissue acquisition, which has limitations such as invasiveness and poor dynamic monitoring ability (6).

The incidence of non-alcoholic fatty liver disease (NAFLD) has been rising in recent years and has become a global public health problem (7, 8). Fatty liver background may alter the ultrasound presentation and perfusion of focal hepatic lesions, complicating diagnostic imaging (9, 10), whereas the effect of NAFLD on intrahepatic tumor vascularization and proliferation is unknown.

Contrast-enhanced ultrasound (CEUS) become an important tool for tumor microvessels due to its advantages of being non-invasive, free of ionizing radiation and capable of real-time assessment (11). Conventional blood pool contrast agents, such as SonoVue, reflect the perfusion characteristics of tissues through time-intensity curve (TIC) parameters, and their correlation with MVD has been demonstrated in clinical studies as well as in animal studies including the VX2 tumor model (12, 13). However, there is still a lack of relevant research on the tumor assessment of CEUS in the context of NAFLD at present.

In this study, we constructed VX2 tumors in normal liver and NAFLD background, respectively, compared the CEUS TIC parameters, explored the correlation between TIC parameters and MVD, VEGF, Ki-67, and PCNA, with the aim of addressing the following scientific questions:(1) whether the NAFLD background influences the microperfusion characteristics of VX2 hepatomas; (2) whether the fatty liver background on VX2 tumor microperfusion whether it further affects tumor angiogenesis and proliferative activity. We hope to provide a relevant imaging basis for monitoring the angiogenesis of liver tumors and the efficacy of antiangiogenic therapy in the context of fatty liver.

## 2 Materials and methods

### 2.1 Experimental animals

A total of 24 healthy New Zealand rabbits weighing 2.5 ± 0.5 kg, male and female were randomly selected from Qingdao Kangda Antibody Biological Technology CO., LTD, China, and randomly divided into the normal liver group (n=12) and NAFLD group(n=12). The rabbits in the NAFLD group were fed a high-fat diet(83% standard feed + 10% lard + 5% maltose + 2% cholesterol) for 8 weeks to induce hepatic steatosis (14). The rabbits in the normal liver group were fed with standard rabbit chow. The feeding conditions were appropriate temperature, humidity and light, and the animals were fed and watered freely. The experimental design and animal handling strictly followed the relevant ethical codes of the Experimental Animal Care and Use Committee (IACUC) to ensure animal welfare, and the consent of the Ethics Committee of Shanxi Medical University was obtained (KYLL - 2023-132).

### 2.2 Molding

**Figure 1:**
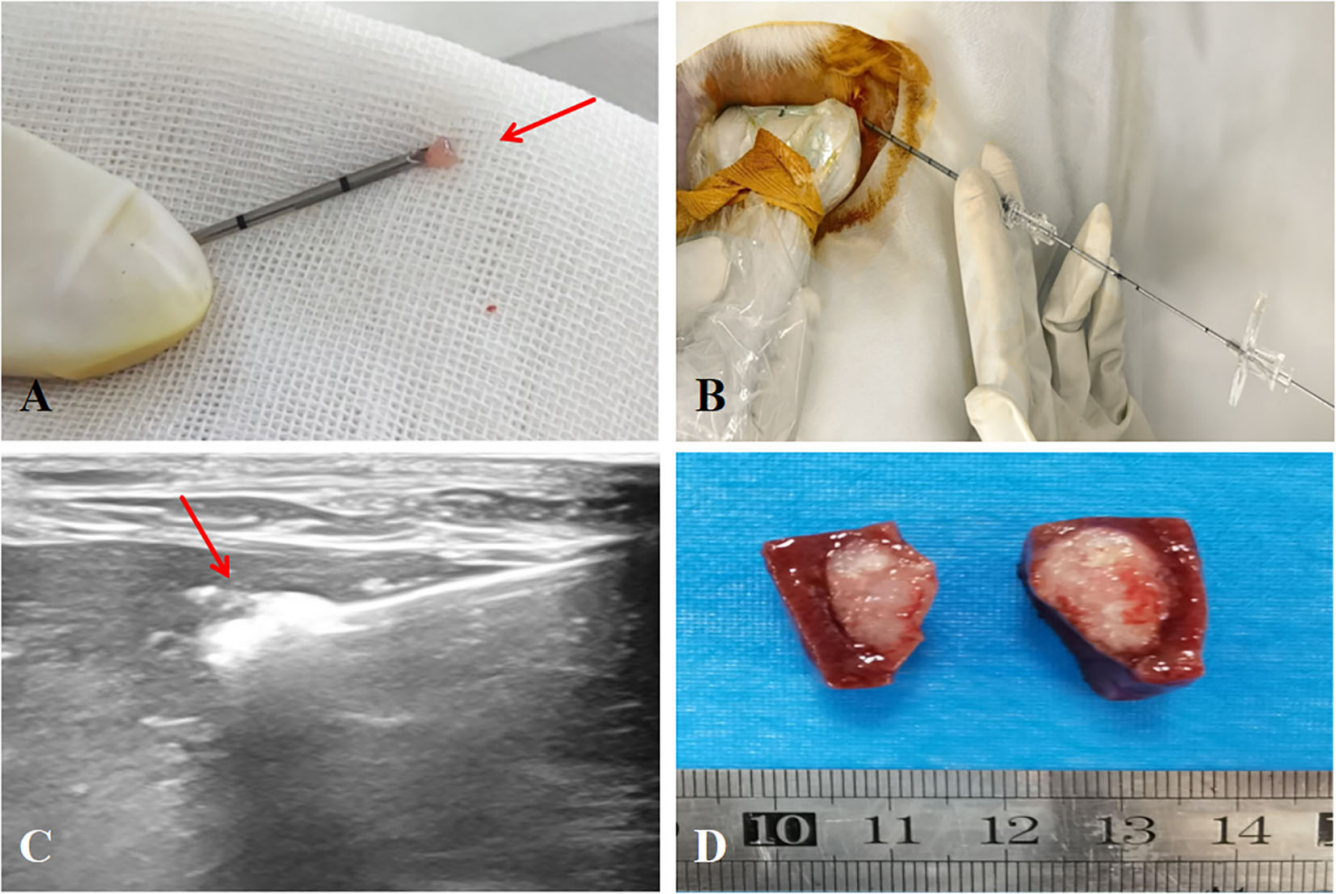
Ultrasound-guided implantation modeling of VX2 tumors in rabbit liver. **(A)** Insert the tumor pieces of 1 - 2mm³ into the needle sheath from the tip(→). **(B)** The needle was inserted into the target area under ultrasound guidance. **(C)** Use the matching needle core to push the tumor pieces in the needle sheath into the liver(→). **(D)** Liver VX2 tumor model.

The VX2 tumor model of rabbit liver was established by percutaneous injection of VX2 tumor fragments under ultrasound guidance. One tumor was implanted respectively in the left liver and the right liver of each rabbit. ① Anesthesia: Anesthesia was induced by intramuscular injection of Zoletil (Virbac, Carros, France) 2 mg/kg (0.04 mL/kg) in the thigh + Sumianxin II (Sheng xin, China) 6 mg/kg (0.06 mL/kg) in the thigh. ② Preoperative preparation: The rabbit was fixed on the experimental table after anesthesia was completed, its upper abdomen was exposed, shaved, the skin was sterilized, and a sterile cavity towel was laid. ③ Preparation of materials: Cut the revived VX2 tumors into small pieces of 1 – 2 mm³. Use ophthalmic forceps to insert 5 – 7 VX2 tumor pieces from the tip of the 18G puncture needle sheath into the needle tube. ④ Ultrasound-guided implantation: The sheath of the 18G puncture needle was inserted into the target liver site under ultrasound guidance, followed by the insertion of the needle core, which pushed out the VX2 tumor pieces in the sheath to the target site. After the implantation was completed, the puncture needle was slowly withdrawn and the puncture point was gently pressed for 2 – 3 minutes to prevent displacement of the VX2 tumor pieces (**Figure 1**).

### 2.3 Ultrasound evaluation

Ultrasound evaluation was performed 14 days after tumor implantation when tumors had grown to an appropriate size (15). Anesthesia and skin preparation are the same as described previously.

A Mindray Resona R9 color Doppler ultrasound diagnostic instrument(Mindray Company, China) equipped with a CEUS module and TIC analysis software was used in this study. The results were recorded after reaching a consensus through consultation between two ultrasound physicians with over 10 years of working experience. Conventional ultrasound examination uses the L15 - 3WU linear array probe to observe the size, shape, echo, margin and color Doppler blood flow signal of VX2 tumors. The classification of blood flow follows the criteria proposed by Adler et al., ranging from 0(no blood flow signal) to 3(multiple reticular or patchy blood flow signals or two clear vessels) (16).

Contrast-enhanced ultrasound: Contrast-enhanced ultrasound uses the L9 - 3U linear array probe. The region of interest (ROI) is placed at the most obvious site of VX2 tumor enhancement to obtain and analyze the TIC curve. Record the enhancement mode and the following quantitative evaluation parameters of contrast-enhanced ultrasound: mean time-intensity curves (mTIC), peak intensity (PI), arrival time (AT), rise time (RT), time to peak (TTP), flowout time (FT), mean transit time (mTT). The contrast agents were SonoVue™(Bracco SpA, Milan, Italy) at a dose of 0.1 mL/kg, administered by intravenous injection at the ear vein.

### 2.4 Pathological examination

The experimental rabbits were euthanized after the experiment. The removed tumor tissues were fixed with 10% neutral formalin, followed by routine dehydration, paraffin embedding and making wax blocks, with a section thickness of 2.5μm. It was stained with hematoxylin-eosin (H&E) and immunohistochemical staining, sealed with neutral gum, and then observed under a microscope.

Immunohistochemical (IHC): The immunohistochemical antibody kit was purchased from Proteintech (Proteintech,Wuhan, China). The immunohistochemical staining operation steps were carried out in accordance with the reagent manual and the reference method. All sections were independently evaluated by two experienced pathologists who were unaware of the experimental groups using the ImageJ software in randomly selected images. If there were differences in the scores, an agreement was reached through negotiation. The MVD count was first conducted under a low-power microscope (×100) to identify the “hot spot” area, and then under a high-power microscope (×400) to count the number of CD34-positive endothelial cells or cell clusters that were stained brownish-yellow or brownish and could be clearly distinguished from surrounding tumor cells and connective tissues. The average value of the three high-power fields was taken as the MVD value of the specimen (17). The expression percentages of VEGF, Ki67 and PCNA were determined by dividing the stained area by the total area of the region. Calculate the percentage of positive staining area in at least six fields for each section (18).

### 2.5 Statistical analysis

Statistical analysis was conducted using OriginPro 2024 software. Quantitative data were expressed as mean ± standard deviation (mean ± SD). Student’s t-test was used for inter-group comparisons, and chi-square test was used for rate comparisons. The correlation analysis between variables was conducted using the Pearson correlation coefficient. The difference is considered statistically significant when the *P* value is less than 0.05. *Post-hoc* power was calculated using G*Power 3.1.9.7 with α=0.05.

## 3 Results

### 3.1 Conventional ultrasound

Ultrasound examination identified 23 tumors in both the normal liver group and the NAFLD group. On two-dimensional ultrasound, VX2 tumors in both groups mostly presented as isoechoic nodules, most of which had regular shapes, circumscribed or indistinct margins, and irregular necrotic areas could be seen in a few nodules. There were no significant differences in the size, echo, shape and blood flow signal of the lesions between the two groups (*P*>0.05) (**Table 1**). Color Doppler flow imaging(CDFI) for some nodules can show point and linear blood flow signals of different degrees within the lesion. Semi-quantitative assessment was conducted based on the Alder blood flow classification, and there was no statistically significant difference in blood flow signals between the two groups (*P*>0.05).

**Table 1:** Tumor characteristics of VX2 in the background of normal liver and NAFLD.

Tumor characteristics	Normal liver VX2	NAFLD VX2	t/χ2	*P*
Number	23	23		
Size(cm)	0.97 ± 0.22	1.13 ± 0.35	1.905	0.063
Echo				
Homogeneous	15	17		
Heterogeneous	8	6	0.411	0.522
Shape				
Oval/round	15	12		
Irregular	8	11	0.807	0.369
Margin				
Circumscribed	7	4		
Indistinct	16	19	1.075	0.300
CDFI				
0	1	0		
1	7	6		
2	10	8		
3	5	9	2.442	0.486
Enhanced pattern				
Overall hyperenhancement	7	9		
Peripheral hyperenhancement	16	14	0.383	0.536

### 3.2 CEUS

**Figure 2:**
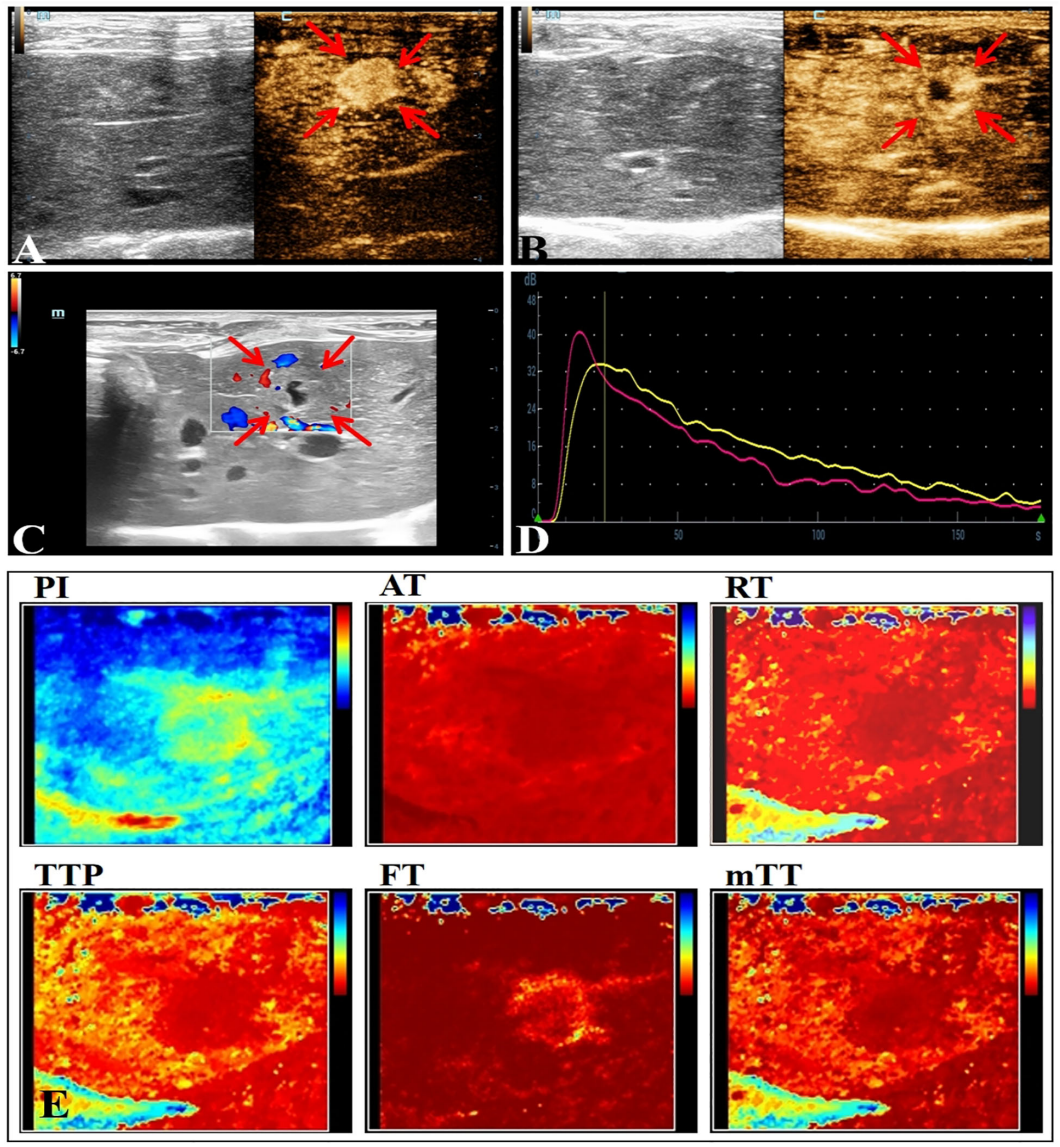
Conventional ultrasound and CEUS manifestations of VX2 tumors in rabbit liver. **(A)** The arterial phase of CEUS shows overall hyperenhancement. **(B)** The arterial phase of CEUS shows peripheral hyperenhancement. **(C)** Ultrasonographic presentation of rabbit liver VX2 tumor with nodular echoes close to the liver, unclear borders, punctate blood flow signals seen within, and irregular necrotic cystic areas seen in the center. **(D)** CEUS TIC curve. **(E)** Visualization image of the CEUS TIC curve parameters of VX2 tumor.

Most of the VX2 tumors in rabbit liver show obvious peripheral hyperenhancement during the arterial phase, overall hyperenhancement in some cases, and hypoenhancement in the early arterial phase (**Figure 2**). The quantitative parameter of CEUS of NAFLD group showed a decrease in AT and RT(*P*<0.05) (**Table 2**).

**Table 2:** Quantitative CEUS parameters of VX2 tumors in normal liver and NAFLD.

CEUS parameters	Normal liver VX2	NAFLD VX2	t	*P*
mTIC(dB)	14.49 ± 6.76	11.85 ± 5.75	1.430	0.160
PI(dB)	30.37 ± 6.92	32.76 ± 7.56	1.119	0.269
AT(s)	9.98 ± 2.00	8.37 ± 1.72	2.919	0.005
RT(s)	11.23 ± 3.25	8.89 ± 2.40	2.770	0.008
TTP(s)	20.27 ± 4.10	18.24 ± 4.12	1.674	0.101
FT(s)	115.60 ± 36.69	122.90 ± 36.68	0.667	0.508
mTT(s)	129.13 ± 36.09	128.87 ± 37.67	0.024	0.981

### 3.3 Pathological results and IHC

Gross specimen appearance: Normal liver tissue appeared dark red with a soft texture. Livers in the NAFLD group were mildly swollen with slightly rounded edges, exhibited a milky yellow color throughout, and showed focal yellowish-white degenerative foci. The cut surface appeared greasy. VX2 tumor nodules were round, white-tan in color, firm in texture, lacked a capsule, and were clearly demarcated from the surrounding liver parenchyma.

**Figure 3:**
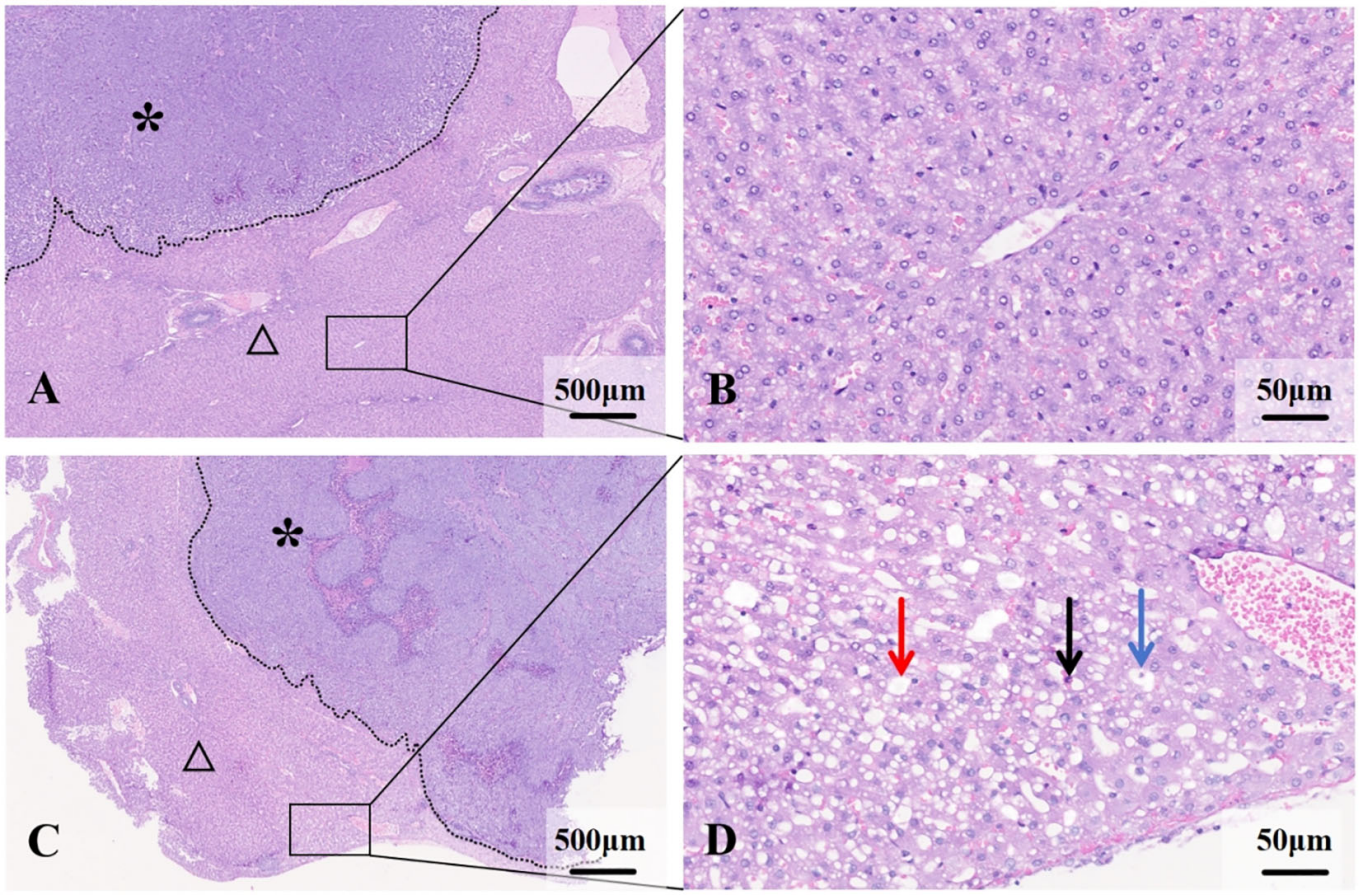
Histological features of liver and VX2 tumors in normal and NAFLD backgrounds. **(A)** VX2 tumor (*) in the normal liver background (△). **(B)** Hepatocytes are normal in size and the hepatic cords are well aligned. **(C)** VX2 tumor (*) in the NAFLD background (△). **(D)** Hepatocytes are swollen and disorganized, with lipid droplets visible within (→), ballooning of hepatocytes (→) and inflammatory cell infiltration (→).

**Figure 4:**
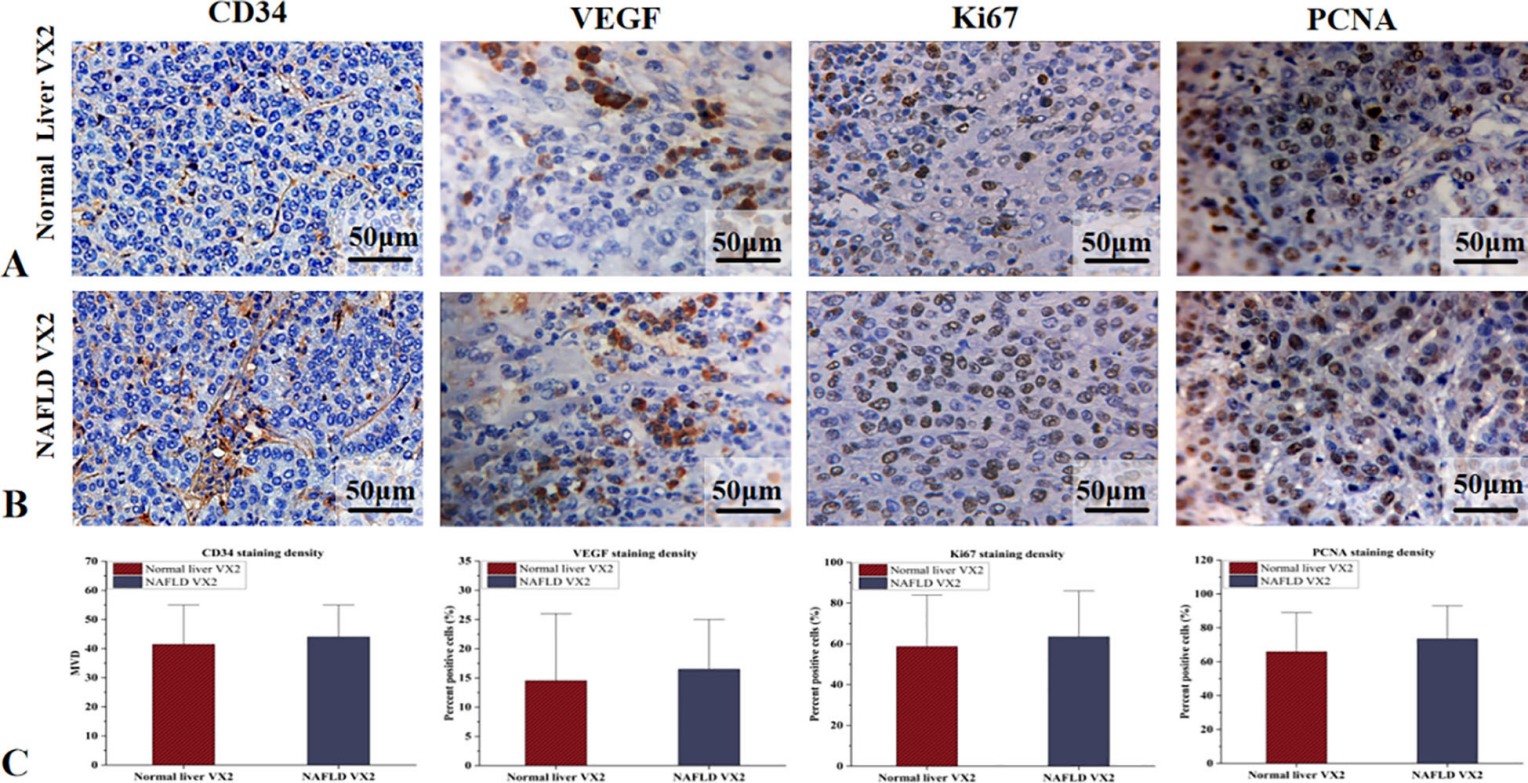
IHC analysis of normal liver and NAFLD VX2 tumors. **(A)** MVD, VEGF, Ki67, and PCNA expression in normal liver VX2 tumors. **(B)** MVD, VEGF, Ki67, and PCNA expression in NAFLD VX2 tumors. **(C)** The differences in MVD, VEGF, Ki67 and PCNA between the two groups did not reach statistical significance.

Microscopy: Liver tissue in the NAFLD group exhibited features consistent with mild to moderate NAFLD. Fatty liver cells were enlarged to varying degrees, arranged in a crowded fashion, with vacuoles of varying numbers and sizes visible in the cytoplasm, narrowed hepatic sinusoids, and foci of inflammatory cells and necrosis were visible in some of the lobules (**Figure 3**).The VX2 tumor cells were arranged in a nest-like fashion, with a large, deep staining, large densely stained nuclei, and nuclear schizophrenic phases were visible. Areas of patchy necrosis were seen within the nodules. Immunohistochemical analysis lacked statistical differences in MVD, VEGF, Ki67 and PCNA between the two groups(*P*>0.05, *post hoc* power=0.15~0.37) (**Figure 4**).

### 3.4 Correlation analysis of CEUS parameters and IHC

**Figure 5:**
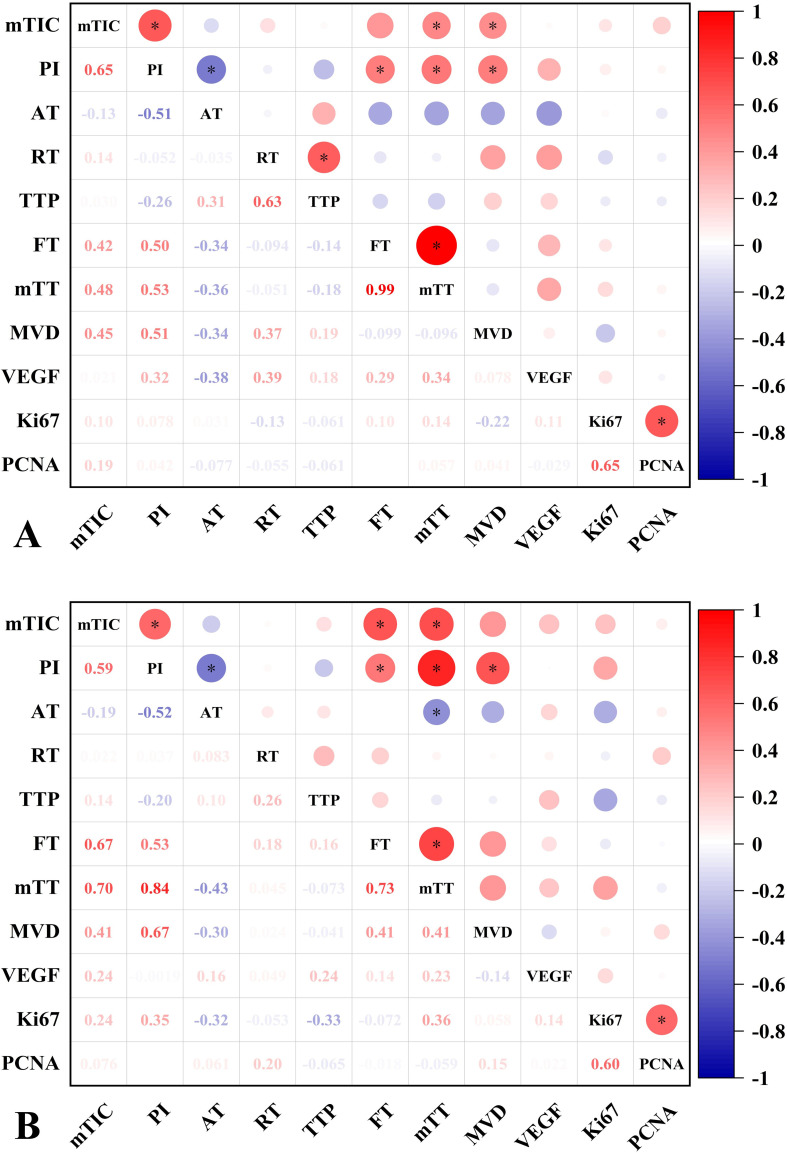
Correlation analysis of CEUS parameters and IHC characteristics of normal liver and NAFLD VX2 tumors. **(A)** Correlation graph of CEUS parameters and IHC characteristics of normal liver VX2 tumors. MTIC and PI were moderately positively correlated with MVD. there was no significant correlation between VEGF, Ki67, PCNA and CEUS parameters. **(B)** Correlation plot of CEUS parameters with IHC characteristics in NAFLD VX2 tumors. PI was moderately positively correlated with MVD. There was no significant correlation between VEGF,Ki67, PCNA and CEUS parameters. Numbers = correlation coefficient. *, *P*<0.05.

In the normal liver group, PI and mTIC were positively correlated with MVD (r=0.51/0.45, *P*<0.05). While, in the NAFLD group, PI was positively correlated with MVD (r=0.67, *P*<0.05), mTIC was positively correlated with MVD (r=0.41, *P*>0.05, *post hoc* power=0.82). There were moderate positive correlations between PI and MVD in all groups. CEUS parameters lacked direct correlation with VEGF, Ki67 and PCNA in both groups of hepatic VX2 tumors (**Figure 5**).

## 4 Discussion

This study explored the alterations in CEUS quantitative parameters in VX2 tumors under the background of NALFD and the correlation between CEUS parameters and pathological features. It was found that the AT and RT of VX2 tumors in the NAFLD group were significantly reduced, and no significant differences in the general characteristics of the tumors in the two groups, including size, morphology, blood flow signals, and immunohistochemical markers MVD, VEGF, Ki67, and PCNA. In addition, the PI of CEUS was positively correlated with MVD in both normal and NAFLD backgrounds, while PI showed no significant correlation with Ki67, PCNA, or VEGF.

### 4.1 Differences between NAFLD VX2 tumors and normal liver VX2 tumors

VX2 tumors in the background of NAFLD showed decreased AT and RT in CEUS parameters. This phenomenon is closely related to the effects of NAFLD on hepatic microcirculation and vascular structure. Studies have shown that fatty liver causes compression and narrowing of the hepatic sinusoids, leading to increased portal pressure and slower blood flow (19), which may trigger a hepatic arterial buffer response (HABR), leading to an acceleration of hepatic arterial blood flow to compensate for the decrease in portal blood flow and to maintain a relatively constant total hepatic blood flow (20). Since hepatic VX2 tumors are mainly supplied by the hepatic artery (21), their blood supply may fill up early and rapidly with increased hepatic arterial perfusion, as evidenced by a decrease in AT and RT. The present study is in agreement with the results of Balasubramanian et al (10) who found that portal blood flow slows down in NAFLD, while hepatic artery flow is compensated for by increased flow velocity. In contrast, Jing Gao et al (22), Claudio Tana et al (23) concluded that portal flow slows down in NAFLD with no significant change in hepatic arterial flow, which is at variance with the results of the present study. However, the alterations in increased flow velocity in the supplying artery were not accompanied by significant elevations in MVD, VEGF, Ki67, and PCNA, suggesting that the alterations in microcirculation in NAFLD VX2 tumors may be driven by changes in perfusion in the liver itself rather than by angiogenesis or proliferation of the VX2 tumors.

### 4.2 The correlation between CEUS parameters and IHC characteristics

CEUS were used to evaluate the microcirculation of VX2 tumors in this study. The CEUS parameter PI were positively correlated with MVD under different liver backgrounds. MTIC showed correlation in the normal liver group. PI represents the enhancement amplitude when the contrast agent reaches its peak in the region of interest, while MVD directly quantifies the density of microvessels in tumor tissues through immunohistochemistry. The positive correlation between the two indicates that CEUS can reflect the characteristics of tumor angiogenesis by dynamically capturing the distribution of the contrast agent in microvessels. This is consistent with the findings of Zhang B et al (24) in renal pelvis urothelial carcinoma, Chen W et al (25) in lung cancer, and Li MH et al (26) in colorectal cancer, confirming the good ability of CEUS PI to characterize MVD.

There was a positive correlation between mTIC and MVD in normal liver group. However, in the NAFLD group, the correlation between mTIC and MVD did not reach statistical significance(*P* > 0.05,*post hoc* power = 0.82), which might be related to random error and sampling error. There are few reports on mTIC parameters. Luo X et al (27) reported that mTIC can be used for early warning of vulnerable plaques, and Jiang B et al (28) reported that mTIC is associated with tissue hypoxia in mouse liver cancer models. However, there are currently no reports on the correlation between mTIC and MVD. We found that mTIC was positively correlated with MVD in control group suggesting that mTIC might be a potential effective indicator reflecting MVD. The above results support CEUS as a reliable tool for non-invasive assessment of tumor angiogenesis, and further verifies the applicability of CEUS technology in assessing MVD in the context of NAFLD.

It is worth noting that CEUS, as a non-invasive imaging technique, can sensitively detect the changes in the microcirculation of VX2 tumors even when the pathological feature of MVD does not change significantly. This capability allows CEUS to overcome the limitation that MVD assessment cannot be used for dynamic monitoring. CEUS may identify changes in tumor microcirculation before conventional pathological changes occur, helping clinicians detect changes in the biological behavior of tumors earlier and evaluate the effectiveness of treatment, thereby providing an important basis for the formulation of personalized treatment plans.

This study analyzed the changes in microcirculation of liver VX2 tumors under the background of NAFLD and compared the correlations between CEUS quantitative parameters and MVD of VX2 transplanted tumors in the backgrounds of normal liver and NAFLD. It revealed that the velocity of the artery supplying blood to the tumor increased under the background of fatty liver, and it was found that PI was related to MVD. The research results provide new evidence for the application of CEUS in the assessment of tumor microcirculation and show the potential value of CEUS in tumor diagnosis and treatment monitoring under the background of fatty liver.

In the context of NAFLD, the reduction of hepatic sinusoidal perfusion may lead to an increase in the release of various angiogenic inducing factors such as HGF, HIF - 1, VEGF, thereby inducing angiogenesis and further promoting tumor growth (29, 30). In this study, although there seemed to be a trend of larger volume and increased tumor volume, MVD, and expression of VEGF, Ki67, and PCNA in VX2 tumors under the background of fatty liver, these differences have not yet reached statistical significance. This may be related to multiple factors, for instance, in NAFLD, macrophages secrete various pro-inflammatory cytokines such as tumor necrosis factor -α (TNF-α), interleukin-1 β (IL - 1β), and IL - 6. These cytokines not only participate in inflammatory responses but also have anti-proliferative effects, thereby creating a pro-inflammatory and anti-proliferative microenvironment that inhibits tumor growth (30, 31). In addition, it should also be taken into account that the easy necrosis of VX2 tumors and the limited observation time window, the relatively short time for the tumor to be affected by the microenvironment of fatty liver, and the possible lag in the changes of pathological indicators (32).

There are several limitations to this study. First, the relatively small sample size is one of the main limitations of this study, which may lead to inadequate statistical power and decreased sensitivity. Second, the inevitable respiratory movements of the animals during the scanning process may also interfere with the image quality and accuracy of parameter measurements. Finally, in view of the fact that VX2 tumors reach their optimal state 2 weeks after modeling, and extensive necrosis often occurs after exceeding this time (21), which affects the assessment results, the observation period was not further extended in this study, which has not yet allowed us to explore the dynamic changes in tumor microcirculation and proliferation status over a longer period of time.

## Data Availability

The original contributions presented in the study are included in the article/supplementary material. Further inquiries can be directed to the corresponding author.
